# Analysis of spatiotemporal distribution, variability, and trends of rainfall in Wollo area, Northeastern Ethiopia

**DOI:** 10.1371/journal.pone.0312889

**Published:** 2025-01-24

**Authors:** Asnake Adane, Birhanu Asmerom

**Affiliations:** 1 Department of Geography and Environmental Studies, Wollo University, Dessie, Ethiopia; 2 Department of Physics (Atmospheric Physics), Wollo university, Dessie, Ethiopia; Euro-Mediterranean Center for Climate Change: Fondazione Centro Euro-Mediterraneo sui Cambiamenti Climatici, ITALY

## Abstract

Ethiopia’s agriculture is mostly dependent on rain, though the rainfall distribution and amount are varied in spatiotemporal context. The study was conducted to analyze the distribution, trends, and variability of monthly, seasonal, and annual rainfall data over the Wollo area from 1981 to 2022. To accomplish this, the study utilized the Climate Hazards Group Infrared Precipitation with Stations version two (CHIRPS-v2) data. Standard Rainfall Anomaly Index (SRA) and Coefficient of Variation (CV) were employed to examine rainfall variability and develop drought indices over southern Ethiopia. The Modified Mann Kendall (MMK) test, Sen’s slope estimator and the innovative trend analysis (ITA) were employed to detect temporal changes in rainfall trends over the study period. The study found that the area experienced considerable rainfall variability and change, resulting in extended drought and flood events within the study period. Results from SRA and CV revealed interannual and seasonal rainfall variability, with the proportions of years below and above the long-term mean being estimated at 56% and 44%, respectively. The MMK test showed that the annual rainfall during the Kiremt (summer-main rainy season) had an increasing trend. On the other hand, rainfall for the Belg (short rain season for the study area) season and the Bega (winter) season showed a significantly decreasing trend (p < 0.05). Results from the innovative trend analysis (ITA) also revealed that the annual and seasonal rainfall trends exhibited different trends in varied magnitude for different stations. On a spatial basis, the eastern and northeastern regions of the study area showed trends of increasing rainfall during the *Kiremt* (JJA). Decision-makers and development planners need to design strategies to mitigate the risks posed by changes in rainfall variability and distribution and enhance community adaptation and mitigation capacities in Wollo, Ethiopia.

## Background

Socio-ecological systems across the world are being disrupted due to climate change and variability. Although climate change is a global issue, different countries, continents, and socioeconomic levels may experience varying degrees of adverse effects from climate change [1]. East Africa is one of the most food-insecure regions in the world and requires humanitarian aid due to the unpredictability of annual and seasonal rainfall and extreme weather events [2]. Rainfall plays a crucial role in determining soil moisture availability and distribution, which is essential for crop production, particularly in sub-Saharan African countries that rely on rain-fed farming systems. The type of crops that can be grown and the associated agronomic management practices depend on the amount and distribution of rainfall [3].

Ethiopia is a country that has been experiencing unpredictable rainfall patterns for different durations. Drought is also a common occurrence in Ethiopia, and it has become more frequent in the last few decades. Wollo is one of the most affected regions in Ethiopia [4]. Limited and irregular rainfall in Wollo is the main factor that affects crop productivity. In most cases, precipitation arrives late in the cropping season or ends early before the crops are mature [5]. Moreover, Wollo is a typical example of Ethiopia’s drought-prone regions characterized by high inter-annual variability and poor rainfall records, resulting in less rainfall than the country’s long-term average [6, 7]. This situation makes the area more vulnerable to change.

Hence, developing credible climate related information is of paramount importance [4]. Some of the challenge in developing credible climate information include lack of quality data and limited availability and accessibility of actionable climate and weather information [7]. Inadequate or fragmented spatial climate data coverage makes it difficult to provide location-specific management advisories to smallholder farmers who are far apart from weather stations with long-term data records [3]. Traditionally, the spatial distribution of precipitation is analyzed using ground observations forming a rain gauge network. In developing countries like Ethiopia, the network of rain gauge stations is sparse, and therefore, the interpolation using point-based rainfall information is subjected to a large uncertainty [2].

Therefore, these countries face the significant challenge of lacking accessible and reliable meteorological datasets. Moreover, the findings of the previous studies on the distribution, trend and variability of rainfall have been inconsistent. For instance, studies by [8–11] found no discernible pattern of annual rainfall across the nation. However, for sites in Ethiopia’s southwest, north, and center, [12] and [13] reported a notable decrease in rainfall from June to September. Since the 1990s, Ethiopia has also seen a trend of declining rainfall, which has significantly impacted the nation’s water supply and agricultural output.

The observed discrepancies may be ascribed to the studies’ extensive dependence on data gathered from meteorological stations situated in a few locations, which is thought to have led to an inadequate portrayal of spatial fluctuations [14, 15]. Moreover, gauge stations are sporadically scattered throughout rural Africa despite the great variation in topography and other biophysical environments. For instance, [16] found that the upper Blue Nile basin in Ethiopia has inter-station distances between rain gauges that are greater than the scales of the rainfall; as a result, the network does not adequately capture the spatial variability of precipitation in the basin. When gauged data is used, temporally incomplete records confuse and introduce uncertainties. Furthermore, the inconsistent results from earlier research [17–19] concentrated on larger geographic scales and neglected to account for local variations.

Although rainfall is a highly heterogeneous variable and Ethiopia has diverse topographic features that significantly influence its spatiotemporal variability [20], few local-level climate studies use satellite data. As a result, there has been insufficient use of high spatial resolution satellite data, which could be used to capture small-scale interactions and local variability. [3] conducted a study at coarse resolution, which may have resulted in an inappropriateness of the localized response to rainfall variability and trend. Hence, a good understanding of local climatic conditions is an important prerequisite to identify and implement appropriate adaptation practices.

This study aims to investigate the spatiotemporal distribution, variability, and trend of rainfall in Wollo. Specifically, our objectives are: (i) to evaluate the spatial and temporal distribution of rainfall, (ii) to examine rainfall variability in Wollo throughout the study period, and (iii) to identify trends in rainfall in the Wollo area. The remainder of the paper is structured as follows: the second section covers the materials and methods used in this study, while the third section presents the results and discussions. Lastly, the fifth section outlines the conclusions we have drawn from our findings and potential areas for future research.

## Materials and methods

### Ethics statement

Animals were not the subject of this study, and nor were any endangered or protected species. Special permits were obtained from NMS of Ethiopia to collect and use station data from the selected areas.

### Description of the study area

**Fig 1:**
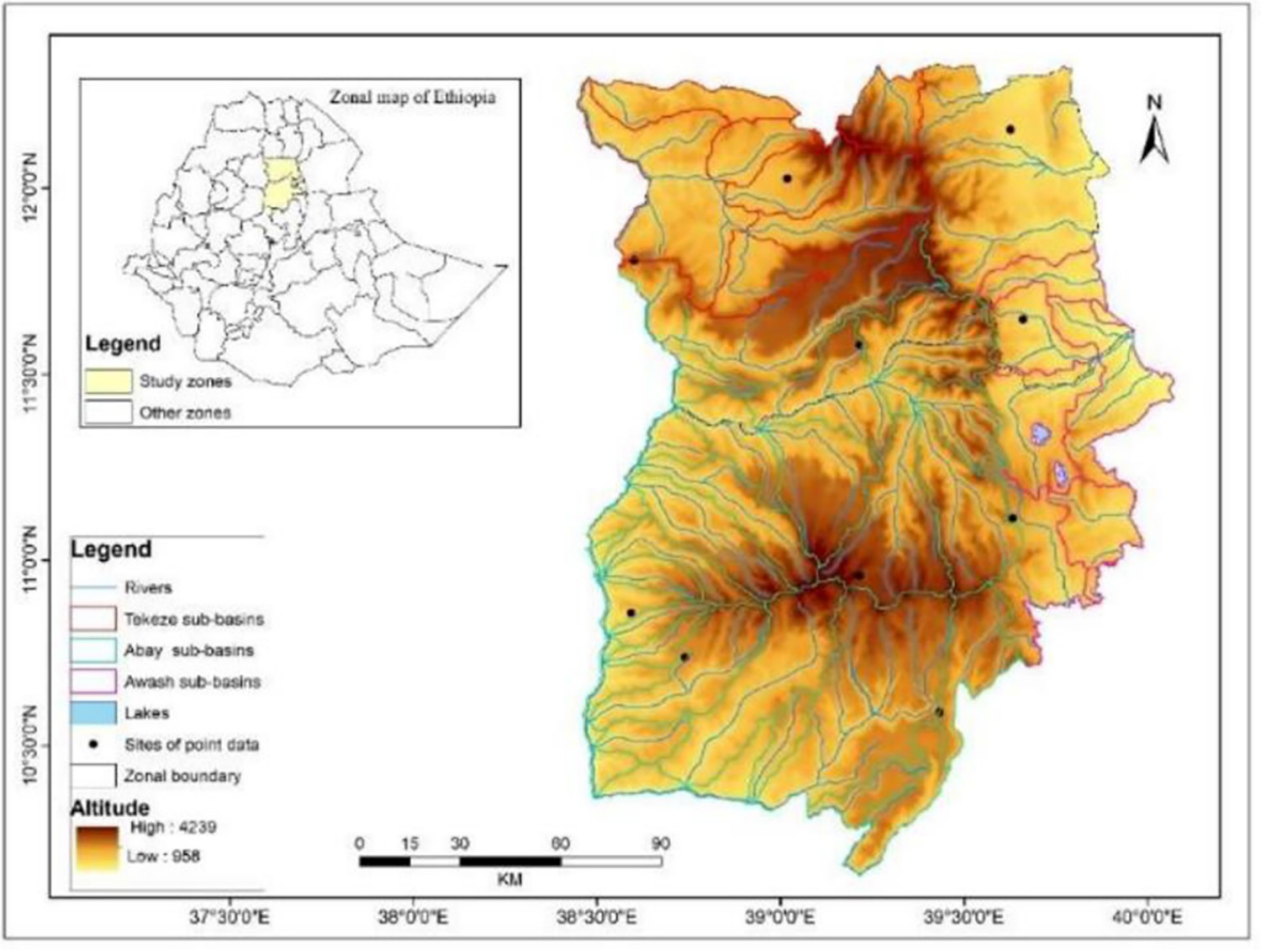
Location of the study area in Ethiopia (shapefile extracted from https://hub.arcgis.com/datasets/esri::world-countries/explore?location=-1.440657%2C0.000000%2C3.00).

The study area, which is between 10^0^00’N and 12^0^15’ N and between 38^0^50’ E and 40^0^11’ E is considered in this study. Administratively, it includes the north and south Wollo zones of the Amhara region (Fig 1) in northeastern Ethiopia. It has a rugged topography, where deep ravines, broad gorges and numerous rivers and streams dissect high mountains. The western part of the study area is primarily a high plateau, a good portion of which lies between 1,800 m and 2,800 m; it is part of the Ethiopia highlands, with peaks rising well over 3,500 meters and high plateau reaching into the alpine zone. It lies on the windward side of the Ethiopian summer rain-bearing winds (equatorial westerlies). The mean annual temperature during the growing period varies from 10 to 15°C.

The eastern part of the study area includes part of the western escarpment of the Ethiopia rift valley, stretching north to south. The topography downslopes eastward, interspersed by a high plateau, until it merges with the Afar lowlands. It also includes scattered graben lands (many groundwater potential sites) that have a genetic relationship with the Ethiopian Rift Valley. The altitude varies from 958 to 4239 m above sea level (Fig 1), and much of the area falls within the *qolla* ecological zone. It lies on the equatorial westerlies’ leeward side, bringing summer rainfall to many parts of Ethiopia. The mean temperature during the growing season may range from 15 to 20°C, and the mean annual precipitation reaches 500 mm.

The up streams and tributaries of Abbay-Awash-Tekkeze River systems drain Wollo (Fig 1). It strides on the water divide of the northwestern drainage system, where streams and rivers from the rugged highlands discharge their contents into the Abbay-Beshilo-Tekkezae river systems in the west and the rift valley drainage system where the Awash River receives the waters of numerous streams.

In a typical year, many parts of Wollo experience two rainfall seasons, and many peasants follow a bimodal farming system. The two seasons are known as *belg* (short rainy season), which fall between mid-February to the end of April, and *meher* (main rainy season), from mid-June to early September, which is responsible for 65%-95% of total annual rainfall in the country [21] (Segele et al. 2015). As a result, the *meher* rainy season is the foundation of agriculture in the Ethiopian highlands and, a key driver of economic development and the primary determinant factor for famines that punctuate the country’s history. The rainfall characteristics in the study area are influenced by several factors ranging from micro- to global scale. These factors include the micro-scale of topography, the meso-scale circulation induced by regional wind systems, and the Inter-Tropical Convergence Zone (ITCZ). The region has high agricultural activity for subsistence and plantation farming. The agricultural activities in this area are mainly under rainfed conditions. During years of good harvest, the average peasant practicing the bimodal system will have just enough food from his *meher* harvest to tide him over to the *belg* season.

### Methods

#### Data type and sources

Climate datasets are mainly measured in two ways viz: Gauge-based observations and Satellite Estimates. Although gauge observations are accurate and trusted measurements at a single point, they suffer from several constraints and deficiencies. These include measurement accuracy, incomplete areal coverage of sparsely populated stations, and deficiencies in data quality [22]. This is particularly evident in developing countries like Ethiopia. For instance, WMO estimates that the observational network systems in Africa are eight times less dense than their recommended level of one station per 26,000 km2. The few stations that do exist are unevenly distributed and suffer from maintenance problems leaving much of the continent unmonitored.

Gridded estimates of climate parameters are considered as potential alternatives to observed data since they provide coverage that is more spatially homogeneous and temporally complete [23]. Thus, this study utilizes Climate Hazards Group Infrared Precipitation with Stations version two (CHIRPS-v2) data, which is obtained through a cloud-based geospatial platform, Google Earth Engine (GEE).CHIRPS is a quasi-global dataset (covering the area between 50° N and 50° S) available from 1981 to the present at 0.05° spatial resolution (∼ 5.3 km), and it is produced using multiple data sources [24]. The CHIRPS data product is developed by the United States Geological Survey (USGS) and the Climate Hazards Group (CHG) at the University of California. We chose the CHIRPS data product because rainfall estimates from this data source in east Africa were found to correlate better with observed data than others like NASA power and TAMSAT [24].

#### Validation of CHIRPS rainfall data

Satellite observations provide broad coverage of global atmospheric parameters with adequate spatial and temporal resolution in un-gauged regions, such as the oceans, complex mountain areas, and deserts [22]. However, they contain non-negligible biases and random errors emanated from the complicated nature of the relationship between the observations, sampling, and deficiencies in the algorithms. Therefore, it would be important to validate and evaluate their accuracy by using other sources of data. Hence, the study used station data collected from stations (Table 1) as reference data. Observed rainfall data for the period from 1981 to 2022 were collected and necessary quality assessments were undertaken using WMO recommended procedures [2].

**Table 1:** Characteristics of in-situ meteorological stations.

S.N	Station (site)	Location	Altitude(M)	District	Location	Years
1	Ajbar	11.25N, 39.25	2178	Saint	Windward	1981–2022
2	Debre Zebit	11.81N,38.58E	2928	Meket	Windward	1981–2022
3	Dessie	11.14N,39.64E	2657	Desie Zuria	Windward	1981–2022
4	Kobo	12.15N,39.63E	1468	Raya Kobo	Leeward	1981–2022
5	Koke Ager	11.15N,39.13E	3456	Legambo	Windward	1981–2022
6	Lalibela	12.03N,39.04E	2500	Bugna	Windward	1981–2022
7	Mekane selam	10.75N,38.76E	1827	Borena	Windward	1981–2022
8	Mersa	11.66N,39.66E	1625	Habru	Leeward	1981–2022
9	Wegel Tena	11.35N,39.13E	3010	Delanta	Windward	1981–2022
10	Wereillu	10.83N,39.16E	3030	Wereillu	Windward	1981–2022

Gridded data for rainfall for the same period were extracted for the grid cells in which selected weather station falls. Scarcity of station data remains a challenging task particularly in developing countries and in remote parts of the world where ground-based precipitation measurements, such as rain gauges are either sparse or non-existent [25]. This is also true for the study area. As a result, this study used point data about these selected locations (whose data are incomplete and/or non-existent) from other gridded data (Terra Climate Data) to enhance the validating role of the station data. Such technique has been employed by previous studies (eg., [26, 27]).

Furthermore, precipitation data from gridded products are known to contain systematic biases relative to station data [28] leading to either over or underestimation of the frequency and/or intensity of the observed precipitation values [23]. Therefore, there is a need to minimize these errors and reduce the differences between gridded and recorded observations using bias-correction techniques. Several bias correction methods, from simple linear scaling methods to complex power transformation and quantile mapping were considered and used to improve the match between observed and derived weather parameters. Comparative assessments of several bias correction techniques has indicated that simple bias correction methods such as linear scaling are as good as complex methods such as quantile mapping [29]. In this study, we employed the linear scaling technique which uses a scaling factor to correct the amount of rainfall. The advantage of this method is its simplicity and modest data requirements. In this method,

$$P_{model,corr}=P_{model}\;X\frac{P_{obs,monthly\;mean}\;}{P_{model,monthly\;mean}}$$ (1)

Where, P_model,corr,_ and P_model_ are corrected and uncorrected monthly rainfall amounts from the model; P_obs, monthly mean_ and P_model_, _monthly mean_ are the monthly mean observed and modelled rainfall amounts.

Previous studies (eg. [30]) used correlation and error analysis to assess the significance of differences between the observed and gridded data sets. However, these techniques can be misleading [27]. This is because the coefficient of correlation is highly sensitive to outliers, hence may not explain the full capacity of a model [31]. To overcome these drawbacks, error matrices like Mean Absolute Percentage Error (MAPE) and the Normalized Root Mean Square (NRMSE) can be used. Hence, we employed MAPE and NRMSE to analyze the overestimations and underestimations from the CHIRPS product. These parameters were calculated for precipitation data before and after bias correction. Below is the mathematical formulation of these error metrics:

$$MAPE=\frac{1}{N}\;\sum_{i=1}^{N}|\frac{X_{obs,i-X_{model,i}}}{X_{obs,i}}|\;X\;100\%$$ (2)

Where X_obs_,_i_ is the actual value for the i ^th^ year, and X_model_,_i_ is the model derived value for the same year and N is the total number of observations.


$$NRMSE=\frac{RMSE}{X_{obs,mean}}$$ (3)

Whereby

$$RMSE=\sqrt{\frac{1}{N}\sum_{i=1}^{N}\;{\left(X_{obs,i}-X_{model,i}\right)}^{2}}$$ (4)

Whereby X_obs, mean_ is the mean of observed data and X_model_,_i_ is the model derived value. RMSE is often expressed as a percentage, where lower values indicate less variance and better match.

#### Data analysis

***i*.** *Analysis of rainfall variability*. Long-term means (LTM) for annual, seasonal, and monthly CHIRPS-v2 rainfall were generated and plotted to visualize spatial-temporal patterns of rainfall in the study area. The difference between total rainfall for each year and the LTM was divided by standard deviation to derive the annual rainfall anomalies. The Rainfall variability was examined using standardized rainfall anomaly (SRA). The anomalies indicate the departure from LTM, with negative values representing periods of below-normal rains (droughts), while positive values reveal above-normal rains (flood risk). The SRA is useful to identify the dry and wet years in the record and addresses droughts that affect agriculture and other livelihoods. The annual and seasonal precipitation averages were used to calculate the rainfall anomaly. The standardized rainfall anomaly was mathematically expressed as:

$$SRA=\frac{Xi-u}{\sigma }$$ (5)

Analysis of spatial temporal variability was also undertaken by calculating a coefficient of variation (CV) for monthly and annual time series using a raster package—this allowed detection of seasonality of monthly and annual rainfall time series. Samy et al. (2019) indicated that the variability of rainfall is classified as CV < 20 (low variability), 20–30 (moderate variability), and 30 (high variability). A CV is specified as:

$$CV=\frac{\sigma }{u\;}x100$$ (6)

CV is the variation coefficient; σ is the standard deviation; μ is the mean rainfall.

*ii*. *Analysis of trends of rainfall*. Hydro-meteorological time series data are characterized by substantial departure from normality [17]. For such data, the non-parametric methods are preferred for detecting monotonic trends because they have higher power than parametric methods. Hence, to see the presence of trends in a month, season, and annual rainfall data, we used non-parametric Mann–Kendall (MK) test statistic and Sen’s estimator test, which are the most used methods for similar purposes [32]. MK test considers the ranks of the observations rather than their actual values. Therefore, it is less affected by the actual distribution of the data and is less sensitive to outliers. In addition, MK was preferred because it is more frequently used for analyzing trends in hydro-meteorological time series datasets compared to the other non- parametric tests [33]. For a ranked set of observations n, X = x_1_, x_3_… xn, the MK trend statistic S was computed using

The Mann-Kendall statistics "S" was computed by the formula:

$$S=\sum_{i=1}^{n-1}\sum_{j=i+1}^{n}\;sgn(x_{j}-x_{i})$$ (7)

Where x_j_ are the sequential data values, n is the data length of the time series, and

$$sgn(x_{j}‐x_{i})=\left\{ \begin{array}{c} 1\;if\;x_{i}>x_{j}\\ 0\;if\;x_{i}=x_{j}\\ -1\;ifx_{i}<x_{j} \end{array}\right.$$ (8)

The Z score was computed to calculate the significance of the trend based on the confidence limits of the standard normal Z. The significance of a trend is calculated by the Z-score using:

$$\left\{ \begin{array}{l} \;o\\ Z=\frac{s-1}{\sqrt{var(s)}},if\;s>0\\ Z=0,\;if\;s=0\\ Z=\frac{s+1}{\sqrt{var(s)}},\;if\;s<0 \end{array}\right.$$ (9)

When Z value exceeds either of the confidence limit lines, it shows a significant trend at a given significance level.

The magnitude or slope of the trend was quantified using the Theil-Sen method [34]. The trend’s strength is proportional to the magnitude of the Mann-Kendall Statistic (large magnitudes indicate a high trend). The significance of trends and Theil-Sen slopes was evaluated at the nominal significance level of p < 0.05. Positive (+) values indicate an increase in constituent concentrations over time, while negative (-) values show a decrease. The Sen’s slope estimator (SSE) was employed to estimate the magnitude of the trends in the time series data. Thus, if a linear trend exists in a time series, then the actual slope of the trend is estimated using a Sen-Theil trend line an alternative to linear regression, combined with the MK test. The slope (T_i)_ of all data sets is calculated as:

$$T_{i}=\frac{x_{j-X_{i}}}{x-k},for\;i=1,2,3\ldots \ldots N$$ (10)

where xj and xk are taken as data values at time j and k (j > k), respectively. The median of these N values of T_i_ is represented as Sen’s estimator of slope, which is expressed as:

Qi={T(N+1)/2,ifNisodd12(TN2+T(N+2)/2),ifNiseven (11)

Sen’s estimator is calculated as Q_med_ = T (N+1)/2 if N appears odd, and it is used as Q_med_ = TN/2 + T(N+2)/2 if N appears even. Finally, Q_med_ was computed by a two-sided test at 100(1 − α) % confidence interval, which is then used to obtain the actual slope through the nonparametric test. A positive value of Qi suggests an increasing trend, and a negative value of Qi offers a decreasing trend in the time series.

The effect of serial correlation was considered by fitting a modified Mann-Kendall (MMK) procedure [35] (Hamed and Rao 1998), mainly when Qi is different from 0. It is possible to correct the variance of test statistics if significant autocorrelation is detected in the first three lags. In this case, the sample data will be de-trended using the following equation.

$$X{’}_{t}=X_{t}-\beta t$$ (12)

where X′ t is the de-trended series, X_t_ is the original time series value at time t, and β is the slope of trend in a sample data. This was checked by using the following equation.

$$r1=\frac{\frac{n}{n-1}\sum_{i=1}^{n-1}\left\lceil x^{\prime }t-E(x^{\prime }t)\rceil \lceil {x\prime }_{t+1}-E({x^{\prime }}_{t})\right\rceil }{\frac{1}{n}\sum_{t-1}^{n}[{x\prime }_{t}-E({x^{\prime }}_{t})]2}\;and\;E({x\prime }_{t})=\frac{1}{n}\sum_{t=1}^{n}{x\prime }_{t}$$ (13)

where r1 is the lag-1 autocorrelation coefficient, n is the number of observations in the de-trended series, and E (X′ t) is the mean of the de-trended series. Then the following equation was used to determine the presence of lag-1 autocorrelation in the data series for further procedure (Zhao et al., 2018).


$$\frac{-1-1.645\sqrt{\left(n-2\right)}}{n-1}\leq r1\leq \frac{1-1.645\sqrt{\left(n-2\right)}}{n-1}$$ (14)

If r1 falls in the above interval, then the time series data sets are independent observations, and the MK test can be applied to the original data set. Otherwise, the data are serially correlated, and then the autoregressive part should be removed from X. The lag-1 autocorrelation coefficient was calculated to remove the auto-regressive component from the de-trended series. When the autocorrelation coefficient of lag-1 was significant, the autocorrelation component was removed from the series using pre-whitening by the following equation:

$$Y_{1}=Y_{t}‐r1Y_{t‐1}$$ (15)

Where Y1 is a series without an auto-regressive part, Yt is the de-trended series, and r1 is the lag-1 autocorrelation coefficient.

### The innovative trend analysis

**Fig 2:**
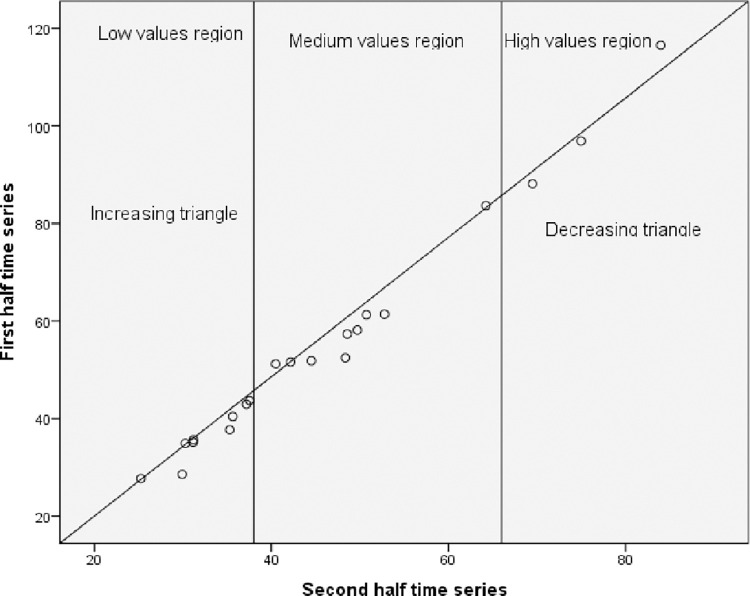
Illustration of the innovative trend analysis (ITA) method.

The hydro-meteorological data that were captured were split into two equal sections, one for the initial time series and the other for the end-time series. Each sub-series was then sorted independently in ascending order. Next, using the two-dimensional Cartesian coordinate system (Fig 2), the first sub-series (xi) was plotted on the horizontal X-axis and the second sub-series (xj) on the vertical Y-axis. The scatter plot’s data points were considered trendless (data without a trend) if they were gathered on the 1:1 (45°) line. A decreasing trend in the time series might be inferred if the data gathered fell into the triangle region beneath the 1:1 line. The time series shows an increasing trend if the data points were above the upper triangle area, or the 1:1 line [26].

The data are categorized into "low," "medium," and "high" value categories in the event of a non-monotonic trend, and the results are displayed in the figure. Consequently, this technique identifies patterns of "low," "medium," and "high" values of any metro-hydrological data series. The percentiles [33] were used to determine the "low," "medium," and "high" value groups in this study based on the amounts of precipitation that were low (X < X − SX), medium (X − SX < X < X + SX), and high (X > X + SX). In these, the first half-time series is represented by X, the mean by X, and the standard deviation by SX. Taking into consideration the pertinent details to be included in this study, the ITA method was applied to the annual and seasonal precipitation data between 1981 and 2022. The ITA method has been applied in recent studies (e.g. Wang et al. [25]) to assess long-term recorded variables (annual and seasonal rainfall) at the eastern China/Yangtze River Delta.

## Results

### Assessment of serial correlation of data

Autocorrelation results(lag-1) for the rainfall at the 10 meteorological stations in the study area are presented in Table 2. As shown, positive serial correlations were obtained for the monthly, seasonal and annual rainfall. The strongest and weakest correlations were found in a few observations. The autocorrelation relative to the upper and lower limits in monthly rainfall were significant at Ajibar(February and September) and Dessie (February and August). In terms of seasonal rainfall, significant autocorrelations were observed during SON at Dessie, and during DIF at Dessie and Mekaneselam. Autocorrelation has also been considered using the Hamed and Rao method in the MK test. These observations were pre-whitened before data analysis.

**Table 2:** R(lag-1) values for serial correlation test (limits are 0.28 and -0.28).

Time series	Ajbar	Debre zebit	Dessie	Kobo	Koke ager	Lalibela	Mekane selam	Mersa	Wegel tena	Wereilu
**Jan**	-0.09	-0.19	-0.07	0.08	-0.06	-0.15	0.08	-0.03	-0.11	0.24
**Feb**	0.29*	0.17	0.32*	0.06	0.26	0.18	0.18	0.20	0.22	0.25
**Mar**	-0.07	0.06	0.02	-0.12	-0.06	-0.14	0.00	-0.23	-0.06	-0.03
**Apr**	-0.12	-0.03	-0.12	0.04	0.02	-0.07	-0.05	-0.09	-0.09	-0.08
**May**	0.13	0.17	0.09	0.00	0.07	0.04	0.07	0.03	0.06	0.13
**Jun**	-0.20	0.04	-0.18	-0.10	-0.17	0.01	-0.13	-0.17	-0.18	-0.16
**Jul**	0.10	0.04	0.10	0.03	0.14	0.02	0.05	-0.01	0.08	0.06
**Aug**	0.24	-0.09	0.29*	0.17	0.27	0.12	0.12	0.30	0.22	0.24
**Sep**	0.32*	0.23	0.25	-0.02	0.21	0.12	0.02	0.05	0.23	0.04
**Oct**	0.07	0.18	0.06	0.13	0.18	0.05	0.30	0.22	0.18	0.09
**Nov**	-0.02	0.15	-0.12	-0.01	-0.09	0.09	-0.06	-0.08	0.05	0.01
**Dec**	-0.06	-0.13	-0.06	-0.10	-0.08	-0.10	-0.24	-0.06	-0.07	-0.10
**SON**	0.30	0.27	0.29*	0.13	0.24	0.11	0.06	0.13	0.21	0.11
**DJF**	0.20	0.17	0.37*	0.03	0.26	0.13	0.35*	0.04	0.16	0.32
**MAM**	0.04	0.01	0.01	0.20	0.00	-0.12	-0.09	0.05	0.02	0.02
**JJA**	0.18	0.00	0.25	0.90	0.19	0.09	0.15	0.20	0.14	0.18
**Annual**	0.14	0.23	0.24	0.20	0.11	0.23	0.09	0.25	0.11	0.11

*Value of serially correlated data, and then prewhitened.

### Validation of CHIRPS dataset

The temporal distribution of rainfall and rainfall seasonality were assessed by contrasting the CHIRPS product’s annual mean rainfall projections with data from actual stations. Despite variations in the total amount of precipitation experienced in various months, the temporal distribution of precipitation throughout the year was accurately depicted in all data sets. The data sets were evaluated according to how well they could represent the variability and quantity of rainfall on a yearly basis (Table 3). The coefficient variation (CV) of the annual average rainfall at the chosen stations ranged from 22.5% at Dessie to 39.8% at Kobo, and it varied from roughly 842.88 mm at Lalibela to 1075.93 mm at Wereilu.

**Table 3:** Station data and gridded annual rainfall amounts (mm) and their coefficient of variation (% in parenthesis) at selected stations (1981–2022).

Location	Station data	CHIRPS data
Ajbar	964.73(29.4)	1008.43(25.6)
Debre Zebit	938.45 (28.9)	926.77(33.4)
Dessie	1000.23(22.5)	1147.87(20.1)
Kobo	870.12(41.8)	695.83 (21.6)
Koke Ager	1006.31 (31.5)	1040.24(39.9)
Lalibela	842.88(28.6)	832.21(25.2)
Mekane selam	1020.67 (27.8)	9962.92 (19.6)
Mersa	979.10 (33.3)	979.60 (13.9)
Wegel Tena	915.31(27.3)	880.35(25.6)
Wereillu	1076.93 (29.7)	1086.12 (31.7)

The gridded data set’s annual rainfall ranges from 695.83 mm at Kobo to 1086 mm at Wereilu while the coefficient of variation (CV) from the same data set ranges from 13.9% at Mersa to 33.4% at Debre Zebit. While the discrepancy is within the lower limits of 0.5–5% for Wereillu, Dessie, Ajbar, and Lalibela, the CV from the station data at Kobo is about double that observed with CHIRPS. The mean monthly rainfall amount from gridded data improved significantly and matched well with observed data after bias correction (Fig 2). The minimum and maximum values of monthly rainfall values from the CHIRPS data sets matched well with the observed data after bias correction.

The statistical indices generated in Table 4 also demonstrate the better fit between the observed and gridded data set. After bias correction, there were 70% of locations with fewer than 20% MAPE values, a considerable improvement according to CHIRPS estimates, as opposed to 40% before bias correction. A minor decline in the error residuals with bias correction has also been noted in the average difference in the NRMSE values between the gridded and observed data sets. The number of places with an NRMSE value of less than 0.2 grew from 3 to 5 or from 30 to 50%, indicating an improvement.

**Table 4:** Error metric values i.e. MAPE and NMRE in JJA (main rainy season) rainfall between station data and CHIRPS data set before and after bias correction.

Location	Before bias correction		After bias correction	
	MAPE (%)	NRMSE	MAPE (%)	NRMSE
Ajbar	75.02	0.23	69.44	0.26
Debre Zebit	31.73	0.27	18.61	0.18
Dessie	28.43	0.19	14.23	0.27
Kobo	40.77	0.25	22.43	0.19
Koke Ager	52.67	0.67	33.02	0.43
Lalibela	49.31	0.93	19.89	0.57
Mekane selam	15.27	0.22	13.12	0.20
Mersa	23.46	0.28	21.45	0.17
Wegel Tena	28.39	0.12	19.03	0.15
Wereillu	14.78	0.14	16.4	0.19

### Spatio- temporal distribution of rainfall in the study area

#### Monthly distribution of rainfall

The spatial distribution of monthly rainfall in the Wollo area from 1981 to 2022 is shown in Fig 4. Most of the study area experiences rain from March to May, a brief but significant rainy season for many areas, and from July to September, the main rainy season with the heaviest rainfall in July and August. During the main rainy season, some areas of the region receive up to 350 mm of rainfall each month.

**Fig 3:**
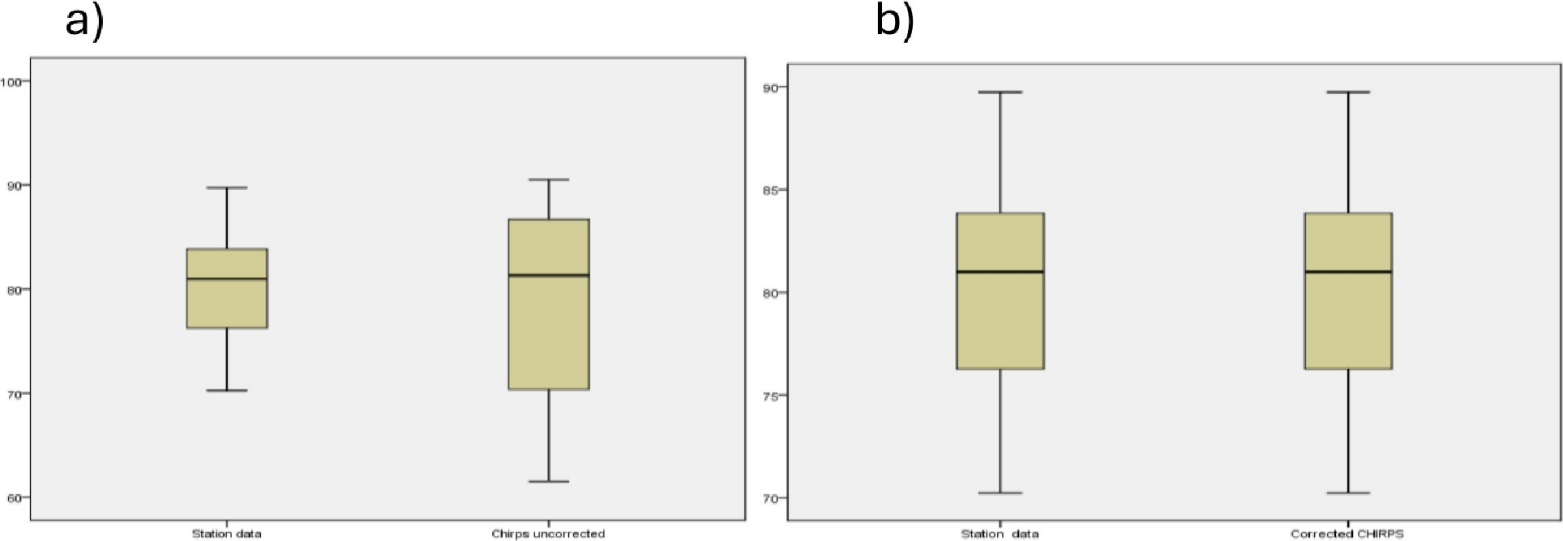
Comparison of box-plot distributions for station and CIRPS data set on monthly average rainfall before correction(a) and after correction(b).

The spatiotemporal distribution of monthly rainfall is significantly influenced by altitude and ruggedness of the terrain. As a result, the study area’s northeastern districts, including Raya Kobo, Habru, Werebabo, and Kalu, continue to lack moisture. On the other hand, the monthly rainfall in the northeast region of the study area decreased during the main rainy season (Fig 3). Since many parts of Ethiopia receive summer rainfall thanks to the equatorial westerlies, which pass through this portion of the study area, topography undoubtedly affects the study area’s rainfall patterns both spatially and temporally. The study reveals that June through August has a high concentration of rainfall, demonstrating the study area’s high temporal variability in rainfall. This suggests that there is a higher monthly as well as inter-seasonal variability in the study area.

#### Seasonal distribution of rainfall

**Fig 4:**
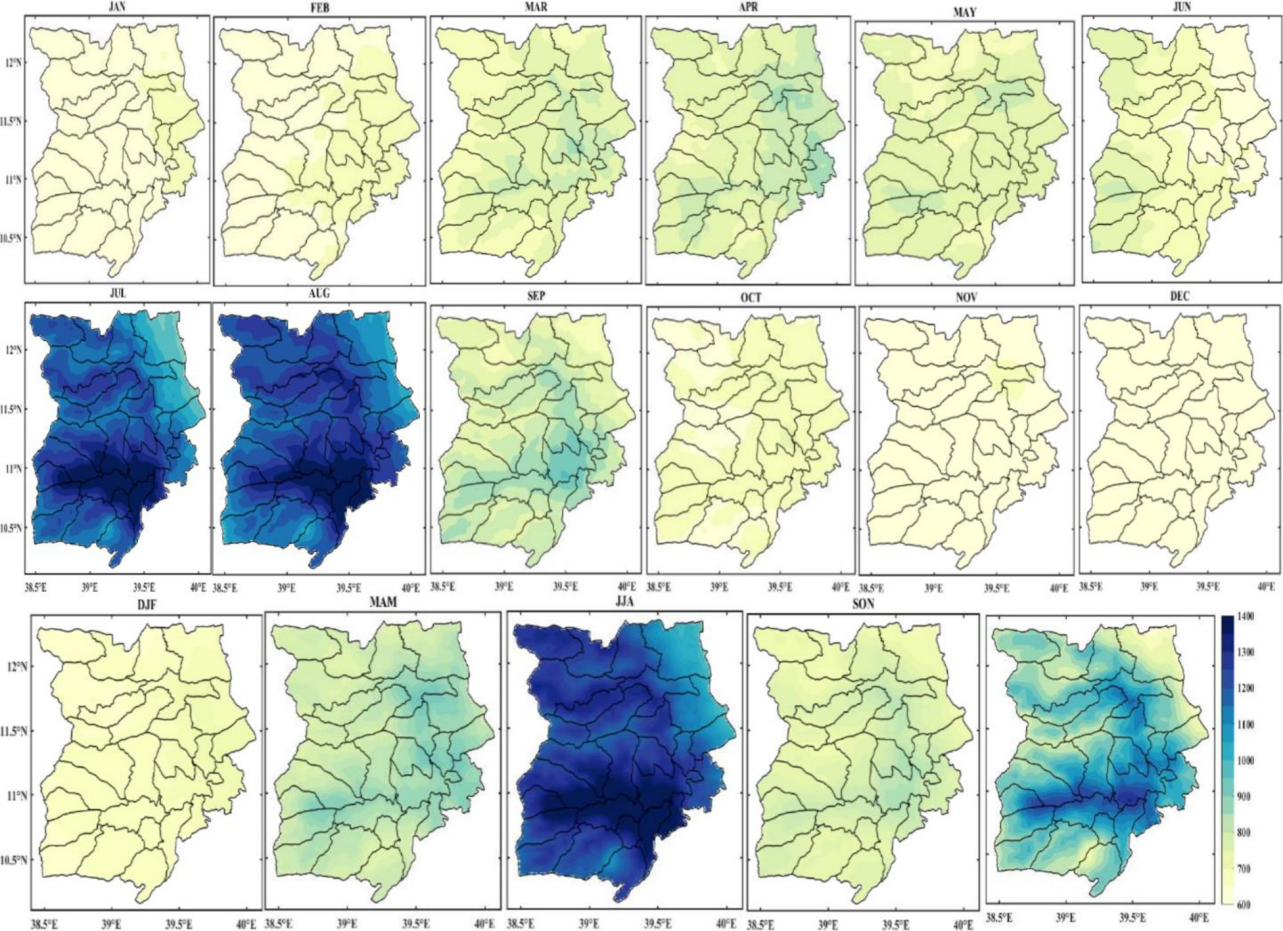
Monthly, seasonal and annual distribution of rainfall in Wollo (1981–2022) (shapefile extracted from https://hub.arcgis.com/datasets/esri::world-countries/explore?location=-1.440657%2C0.000000%2C3.00).

Fig 4 also illustrates how the study area was wet in two distinct seasons: main rain (June, July, August, September, and October) and short rain (March, April, and May). The dry season lasts from November through February, based on the season. The short rain season, which runs from March to May, and the main rainy season, which runs from June to September, could thus be identified for the study area. For many of the study area’s highland regions, the brief rainy season has a decisive effect on agricultural output, especially during the *belg* production period. Approximately half (49.82%) of the yearly rainfall is caused by *belg* rain. Spatially, the study area’s eastern regions saw more rainfall (200–250 mm) during the *belg* season (Fig 4), while the northwest and a small area in the south saw less rainfall (less than 100 mm). This could be because the eastern portion of the study area faces the south-easterlies, which can bring light rain to some areas of the Afar region. It could also be because the east part of the study area is geographically close to the Afar region. On the other hand, the leeward side of these regions from these seasonal winds may cause reduced rainfall in the northwest and southern sections.

The central and western regions of Wollo experienced high rainfall (300–350 mm) during the Kiremt season, while the northeastern region of Wollo experienced low rainfall (<100 mm). Rainfall in the southern and eastern regions was significantly less than in the other parts. This highlights a significant amount of spatial variation in the monsoon rainfall distribution throughout the area. In addition, JJA is the study area’s wettest three-month season, which significantly impacts people’s livelihoods.

The study area, Wollo, is heavily reliant on the short rains which occur between March and May for their entire yearly food production. However, this also makes them more vulnerable to crop failure and drought. The short rains are important for producing food, raising grass, and weed control during the main rainy season. Although short rains only account for 5–10% of the country’s food production, they play a significant role in supporting the livelihoods of those in Wollo.

It is important to note that there is little correlation between the amount of short-term and long-term precipitation in the area. This means that drought can still occur even after good short-term rainfall during the main rainy season, highlighting the high temporal variability and unpredictability of the rain in Wollo at different time scales. The average annual rainfall distribution in Wollo also varies, ranging from 600mm in the northeastern part of the study area to 1400mm in the southeastern and central parts.

### Spatio-temporal variability of rainfall in the study area

#### Monthly variability

**Fig 5:**
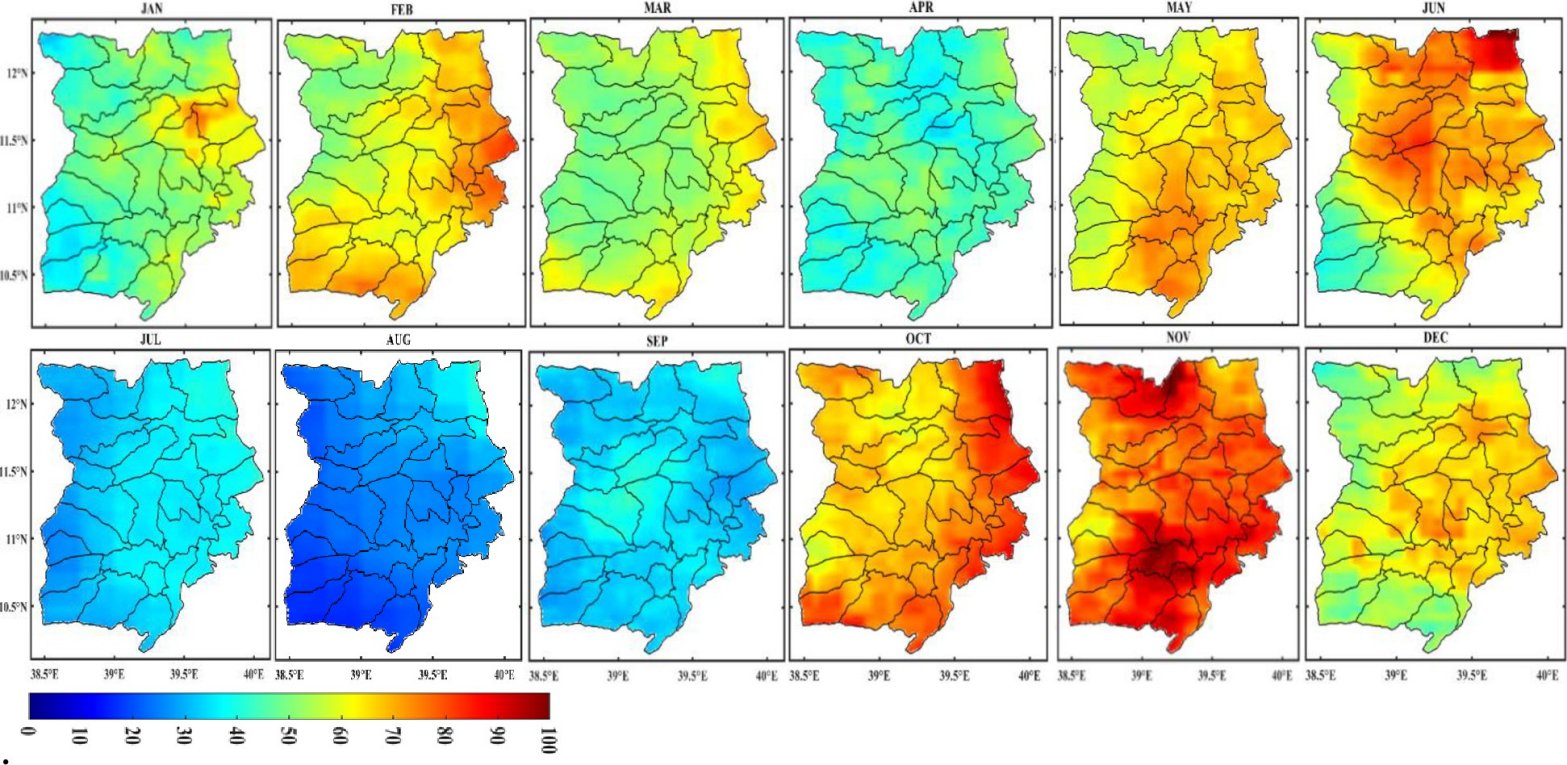
Coefficient of variation (CV) monthly rainfall in Wollo (1981–2022) (shapefile extracted from https://hub.arcgis.com/datasets/esri::world-countries/explore?location=-1.440657%2C0.000000%2C3.00) seasonal rainfall variability.

In Wollo, Ethiopia, monthly variability of rainfall data over 42 years (1981–2022) was examined and shown in Fig 5. Throughout the years under review, there was a great deal of variation in the rainfall distribution. The Figure shows less variation in rainfall in July, August, and September, while there was significant variation in rainfall in the remaining months.

**Fig 6:**
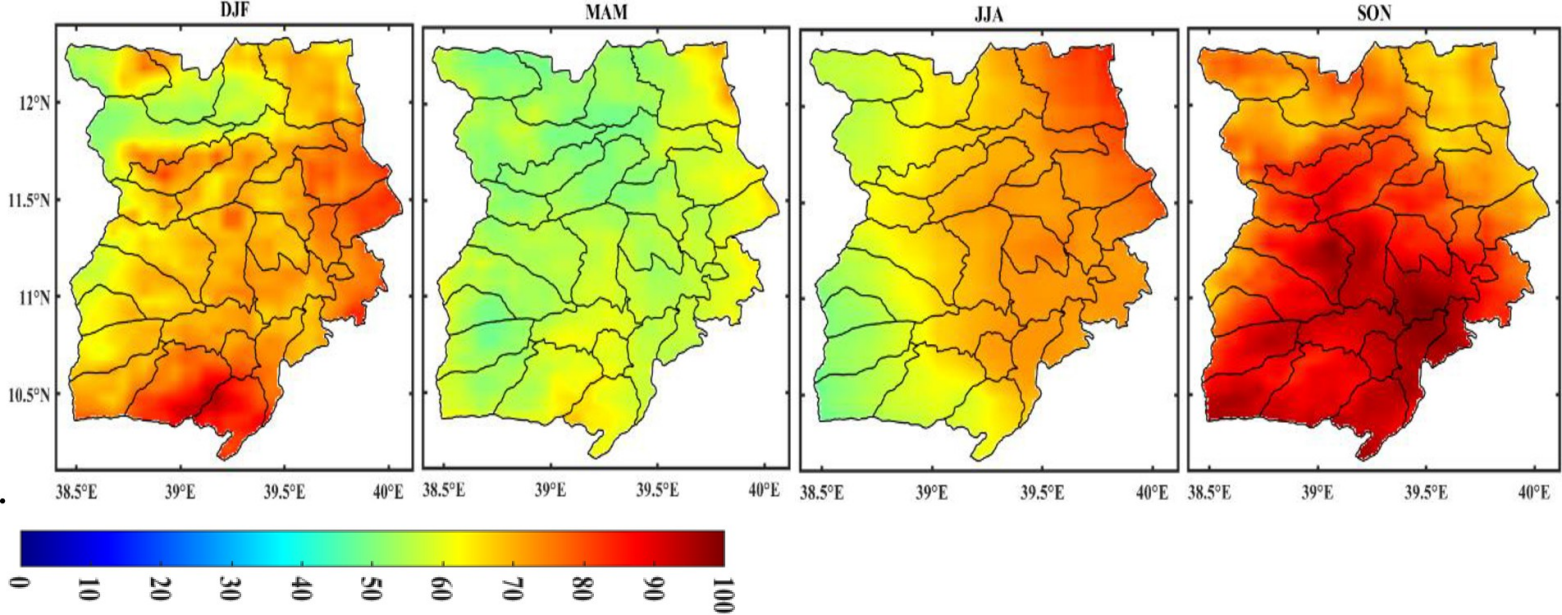
Coefficient of variation (CV) seasonal rainfall in Wollo (1981–2022) (shapefile extracted from https://hub.arcgis.com/datasets/esri::world-countries/explore?location=-1.440657%2C0.000000%2C3.00).

The analysis of the seasonal rainfall coefficient of variation (CV) during the observation period (1981–2022) revealed that the meher (SON) and Bega (DJF) seasons had high CVs of 100% and 83%, respectively. In contrast, the Belg (MAM) and kiremt (JJA) seasons had lower CVs of 52% and 61%, respectively (Fig 6). The analysis also showed that the southeast part of the study area had a greater CV during DJF compared to the northern and northwest regions. Similarly, the eastern part of the study area had a greater CV during JJA than the western portion, with a rising pattern towards the northeast edge of the study area. This can be attributed to the highlands of Ethiopia receiving summer rainfall due to the region’s location on the rain-leeward side of the equatorial westerlies and Guinea monsoon winds. Geographic features of a place can influence the spatial distribution of rainfall in a specific way. This suggests that variations in the aspect, altitude, and direction of the dominant equatorial westerlies and southeastern trade winds are responsible for the spatial variation in rainfall distribution at different spatial scales in many areas of Ethiopia.

Furthermore, the chart in Fig 6 illustrates how the coefficient of variation changes across the different seasons of the study area, indicating that rainfall varies at different spatial scales. Specifically, the meher (SON) season is when the study area experiences the greatest variability in rainfall. During this season, the southern region has the highest variability, which gradually decreases towards the northern and northeastern parts. However, the study area experiences a decrease in rainfall during this season, from the south to the northeast, as shown in Fig 4. These findings suggest that the patterns of rainfall variability and quantity in a particular area may be consistent across different regions. The roughness and terrain characteristics of a landscape typically impact the distribution of drought and rainfall on an annual and seasonal basis.

#### Annual rainfall variability

**Fig 7:**
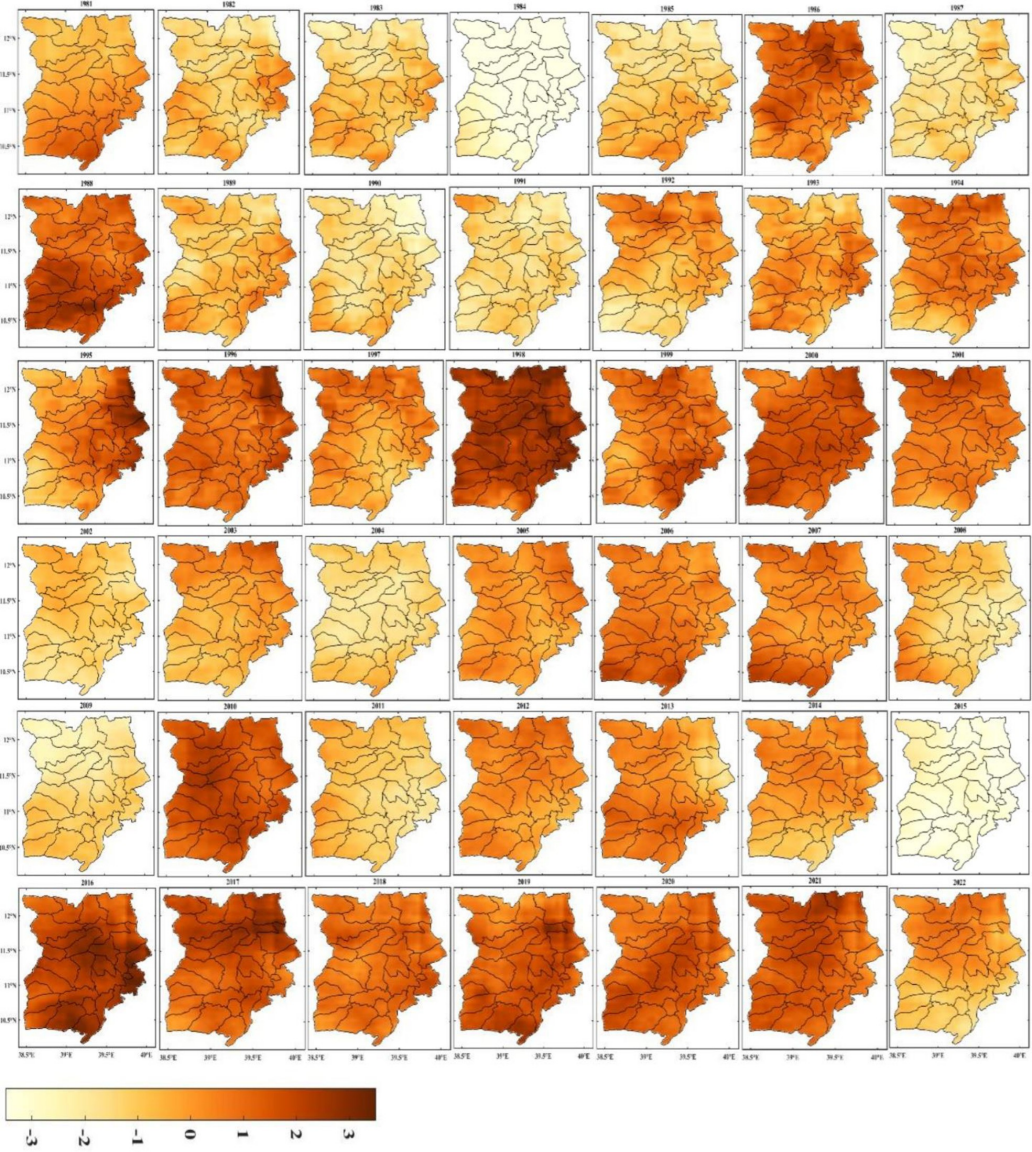
Annual standardized rainfall anomaly (1981–2022) (shapefile extracted from https://hub.arcgis.com/datasets/esri::world-countries/explore?location=-1.440657%2C0.000000%2C3.00).

Fig 7 shows the yearly rainfall anomalies in the study area between 1981 to 2022. The standardized rainfall anomalies reveal that there were positive as well as negative anomalies, indicating the existence of inter-annual rainfall variability throughout the observation period. The most significant negative anomaly was observed in 1984, which adversely affected the primary means of subsistence for rural residents in Ethiopia, particularly in the north. Wollo, as mentioned previously, is one of the drought-prone regions in Northern Ethiopia, and there is significant spatial variability in drought incidents in the area.

During the observation period, the annual rainfall anomalies demonstrated that the region received significantly less rainfall than usual during many years (Fig 7). Droughts repeatedly affected Wollo, resulting in significant crop failure and harvest losses. The figure illustrates the situation during the significant drought years of 1983, 1984, 1992, 1993, 1995, 1999, 2000, 2004, 2009, 2011, and 2015. 1984, 1999, 2009, and 2015 were the four years with the worst droughts.

The primary causes of variations in drought incidences are the topographic aspect, ruggedness of the terrain, and altitude. The occurrences of drought follow the rainfall distribution pattern, as shown in Fig 4. The results of the spatiotemporal analysis indicate that the northeastern and eastern regions of the study area, which are located on the leeward side of Ethiopia’s main summer rain-bearing winds, are more frequently affected by severe and extreme droughts. This suggests that the aspect and ruggedness of the terrain affect the temporal and spatial distribution of rainfall. Fig 9 also demonstrates that only the *kiremt* season (June, July, and August) has rainfall above average, indicating that the study area’s annual rainfall is concentrated in just a few months of the year.

**Fig 8:**
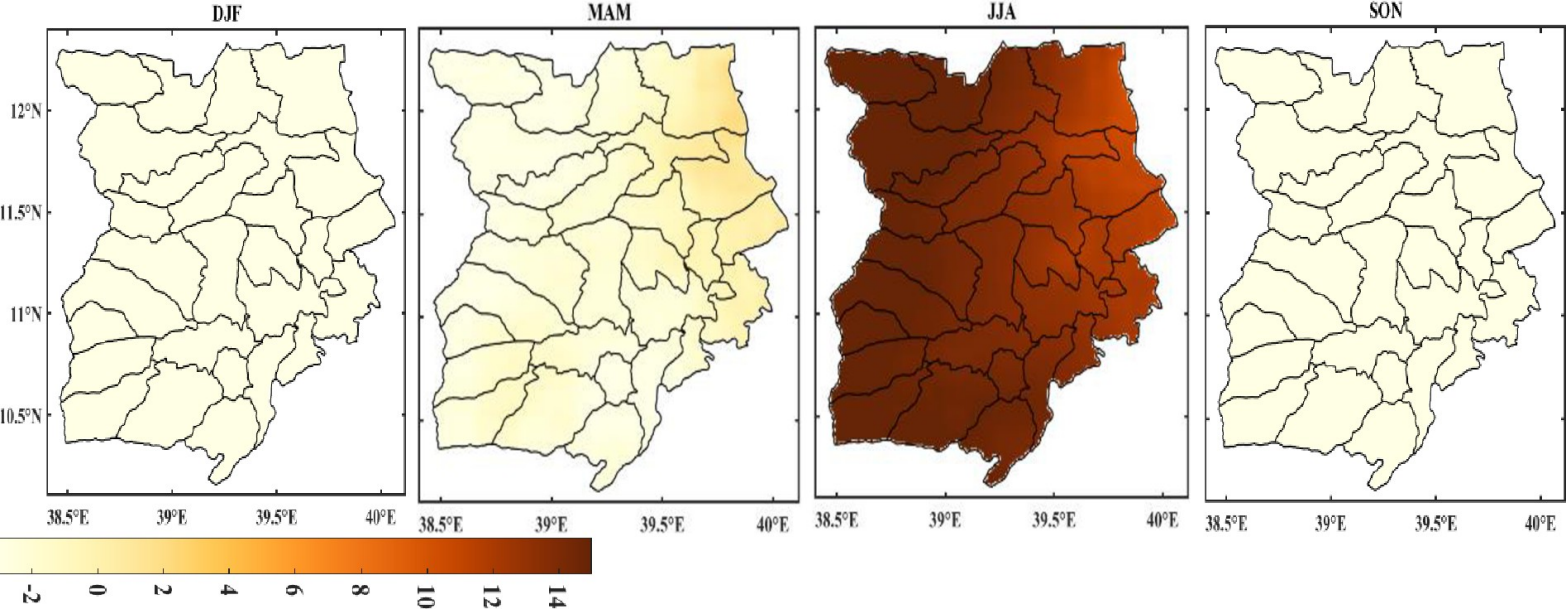
Seasonal standardized rainfall anomaly (1981–2022) (shapefile extracted from https://hub.arcgis.com/datasets/esri::world-countries/explore?location=-1.440657%2C0.000000%2C3.00).

In the past few decades, Ethiopia has experienced several severe droughts that have had significant impacts on the country’s agriculture and food security. For instance, in 1999, the Wollo area was hit by a severe drought that resulted in food shortages. Additionally, other regions also experienced harvest failures during the same year due to extreme drought conditions. In 2008/2009, there was almost a complete crop failure in Belg. The Wollo area was also impacted by droughts in 2009 and 2015-2016(Fig 8), leading to decreased output for many nearby farms.

A report by [36] highlighted the occurrence of severe droughts in Ethiopia’s northeastern highlands during the Kiremt season in 1984 and 2009. [37] further noted that drought incidents in 1984 led to below-average Kiremt season rainfall for an extended period, with northern Ethiopia suffering the most destruction.

### Analysis of rainfall trends

#### Patterns and trends of monthly rainfall in the study area

The study conducted the Mann-Kendall test to determine if the monthly rainfall data sequence exhibits a statistically significant monotonic increasing or decreasing trend. The results showed that a significant trend could be observed at the 0.05 significance level for four months—February, March, July, and August. Kendall’s test results for February and March indicated negative values, implying a decreasing trend in monthly rainfall, while the test results for July and August showed an increasing trend.

**Fig 9:**
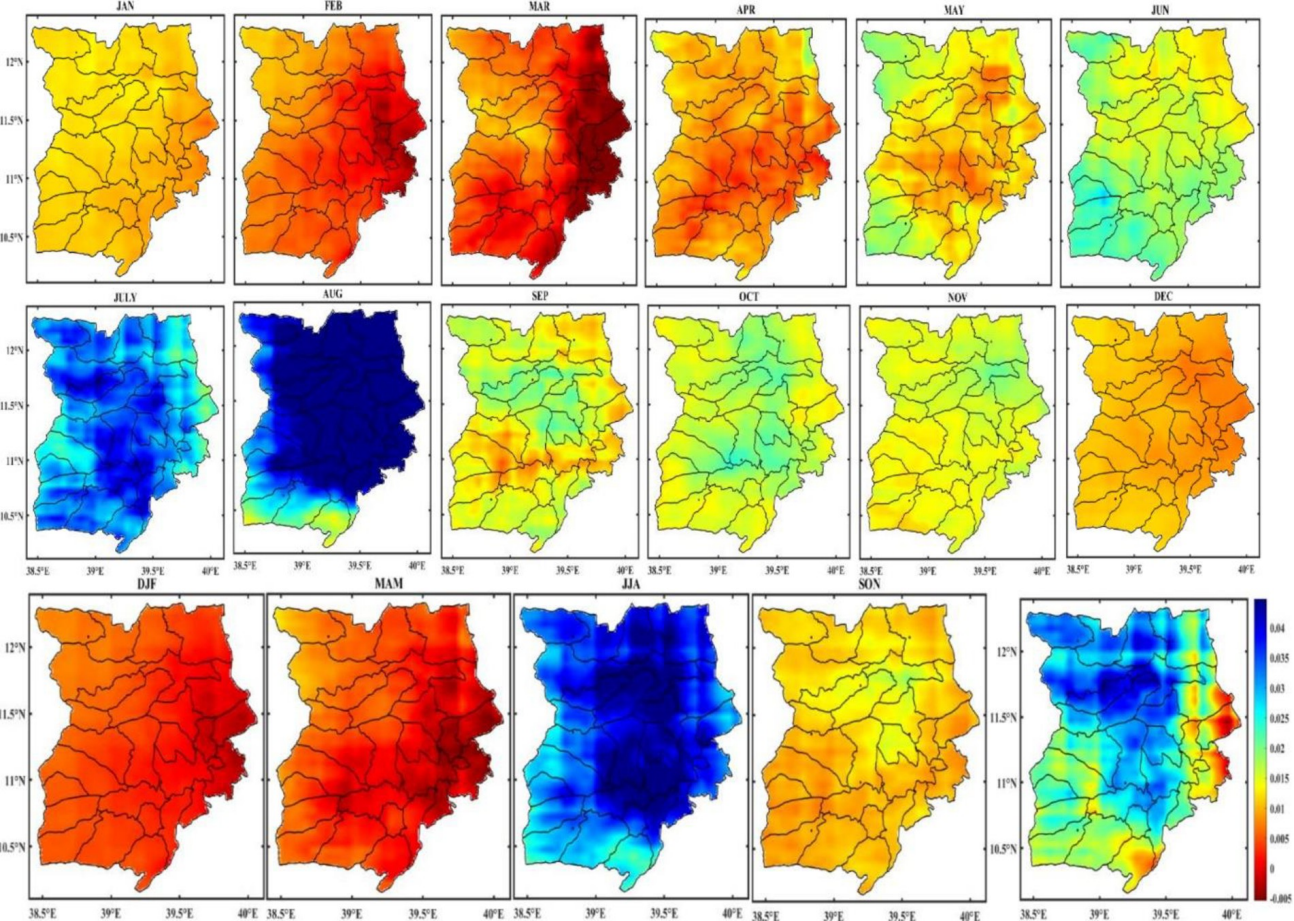
Trends of monthly, seasonal and annual rainfall (mm/year) in Wollo (1981–2022) (shapefile extracted from https://hub.arcgis.com/datasets/esri::world-countries/explore?location=-1.440657%2C0.000000%2C3.00).

During the study period from 1981 to 2022, a decreasing trend was generally observed in the monthly rainfall trend, with some months exhibiting no discernible trend. In terms of space, June, July, and August showed an increasing trend, and statistically significant trends were noted in the study area’s northeastern, southwesterly, and southern regions, as shown in Fig 9. Moreover, a 3.12 mm/year increase was observed in these months according to the Mann Kendall (MK) test. In contrast, the Belg season months (MAM) exhibited a significant (p < 0.05) declining trend.

#### Trends of seasonal rainfall in the study area

Fig 9 illustrates the seasonal trends found in the Mann-Kendall trend test. For the *Belg* (MAM), *Kirem*t (JJA), *Meher* (SON), and *Bega* (DJF) seasons, the seasonal rainfall variability was 0.2 mm/year, 0.6 mm/year, 0.1 mm/year, and -0.2 mm/year, respectively. The trend for Kiremt months (JJA) is increasing while the decreasing trend confines to the Belg rainfall, which was found to be significant at the p < 0.05 level. During the observation period, significant variation has been seen between the seasons (Table 5).

**Table 5:** Results of the Sen’s slope estimator and MKz test for the monthly, seasonal and annual rainfall at the selected stations.

Time	Ajibar	Debre Zebit	Dessie	Kobo	Koke Ager	Lalibela	Mekane selam	Mersa	Wegeltena	Wereillu
Jan	-0.00 (0.98)	0.04 (0.43)	-0.06 (0.43)	-0.01(0.77)	-0.01(0.93)	0.03(0.64)	-0.01(0.74)	-0.07(0.68)	0.04(0.50)	-0.10(0.25)
Feb	-0.42(0.02) *	0.02 (0.81)	-0.44(0.10)	-0.17(0.23)	-0.45(0.02) *	-0.14(0.21)	-0.18(0.17)	-0.55(0.04) *	-0.33(0.05)	-0.25(0.13)
Mar	-0.01 (0.85)	-0.07(0.78)	-1.10(0.08)	-0.51(0.14)	-0.36(0.51)	-0.27(0.42)	-0.25(0.17)	-0.78(0.17)	-0.02(0.96)	-0.40(0.26)
Apr	-0.01 (0.81)	0.11(0.65)	-0.16(0.69)	-0.10(0.79)	-0.30(0.57)	-0.02(0.96)	-0.25(0.53)	-0.34(0.57)	-0.23(0.50)	-0.20(0.71)
May	0.15(0.73)	0.45 (0.31)	-0.16(0.69)	0.00(1.00)	0.13(0.74)	0.20(0.51)	0.33(0.58)	0.07(0.96)	0.15(0.63)	0.30(0.68)
Jun	0.45(0.04) *	0.58(0.03) *	0.36(0.02) *	0.12(0.05)	0.56(0.00) *	0.31(0.13)	0.87(0.01) *	0.3 (0.01) *	0.34(0.01) *	0.55(0.00) *
Jul	1.70(0.2)	1.01 (0.37)	1.65(0.26)	1.17(0.18)	1.83(0.24)	1.18(0.20)	2.13(0.05)	1.57(0.13)	1.42(0.23)	2.40(0.16)
Aug	2.20(0.03) *	1.33(0.07)	2.79(0.01) *	2.40(0.02) *	2.17(0.01) *	2.45(0.01) *	1.26 (0.01) *	2.43(0.02) *	2.07(0.01) *	1.90(0.04) *
Sep	0.44(0.44)	0.46(0.07)	0.38(0.50)	0.09(0.61)	0.14(0.78)	0.50(0.01) *	0.16(0.63)	0.27(0.39)	0.32(0.51)	-0.17(0.68)
Oct	0.20(0.04) *	0.24(0.07)	0.23(0.07)	0.16 (0.00) *	0.23(0.13)	0.18(0.03) *	0.22(0.05) *	0.24(0.05) *	0.18 (0.09)	0.21(0.24)
Nov	0.14(0.01) *	0.22(0.03) *	0.12(0.00) *	0.13(0.04) *	0.12(0.02) *	0.13(0.05) *	0.09(0.09)	0.21(0.09)	0.20(0.02) *	0.15(0.01) *
Dec	-0.02 (0.66)	0.05(0.30)	-0.03(0.66)	-0.02(0.44)	-0.04(0.17)	0.01(0.77)	0.01(0.31)	-0.06(0.39)	-0.01(0.81)	-0.02 (0.29)
JJA	4.7(0.02) *	3.23(0.03) *	5.00(0.01) *	4.38(0.02) *	4.98(0.02) *	4.58(0.01) *	4.41(0.01) *	4.75(0.06)	4.20(0.02) *	4.02(0.05) *
SON	0.8(0.20)	0.84(0.09)	0.91(0.28)	0.43(0.12)	0.29(0.55)	0.68(0.02) *	0.55(0.24)	0.94(0.05) *	0.58(0.11)	0.42(0.52)
DJF	-0.5(0.05) *	0.04 (0.74)	-0.89(0.02) *	-0.42(0.06)	-0.57(0.02) *	-0.10(0.48)	-0.17(0.30)	-0.73(0.04) *	-0.31(0.19)	-0.36(0.11)
MAM	-0.6(0.46)	0.44(0.61)	-0.97(0.21)	-0.56 (0.30)	-0.95(0.38)	-0.09(0.92)	-0.32(0.80)	-1.41(0.23)	-0.15(0.85)	-0.73(0.36)
Annual	4.3(0.06)	4.8(0.00) *	2.96(0.11)	2.90(0.15)	4.14(0.04) *	4.73(0.01) *	4.47(0.01) *	3.24 (0.06)	4.09(0.04) *	3.88(0.04) *

Note: 1. The autocorrelation has also been considered using the Hamed and Rao method.

2. Values in the parentheses are the MKz test values at 0.05% confidence interval

The annual production in drought-prone areas like Wollo is significantly dependent on the short rainy season, which occurs between March and May. However, this over-reliance on short rains makes these areas more vulnerable to climate change. The short rains play a crucial role in producing food (accounting for 5–10% of the country’s food production), promoting early growth that can be plowed back into the ground and raising grass, which helps livestock gain weight after the dry season Unfortunately, there is little correlation between the short rainy season and the primary rainy season, which could lead to a good short rainy season followed by drought during the primary rainy season. This is a common problem in Wollo and other parts of Ethiopia. Interestingly, low rainfall in one area during the primary rainy season may coincide with good rains in another. Therefore, the worst years of drought do not always happen at the same time in different parts of the country.

Although the belg season’s (MAM) rainfall is crucial to the livelihoods of many people in the study area, the distribution and amount of rain in the area are highly variable, contributing to several drought incidents. The rainfall during the Belg season is vital for farmers in many areas of the study area. The declining rainfall trend impacts the study area’s agricultural activities, as the Belg is the primary rainy season in many areas. The long-term rainfall data’s trend analysis generally reveals that the Bega (DJF) and Belg (MAM) rainfalls declined over time. On an annual basis, Fig 9 showed the spatial pattern of the trend of mean annual rainfall. As can be seen from the Fig., the eastern and southwestern parts of the study area exhibit a significantly decreasing trend. On the other hand, the central and northern parts show increasing trends with a statistically significant level.

Table 5 shows the MK test results of different stations at monthly, seasonal and annual time scales. For example, Ajibar exhibited significant decreasing trend in the month of February and in the season of DJF; it had increasing trend in the month of of Jine, October and November as well as the JJA season. Similarly, Debrezebit saw significantly increasing trends during the months of June and November as well as during the season of JJA and the annual rainfall. Moreover, the annual rainfall saw significant increasing trends for Kokeager, Lalibela, Mekaneselam, Wegeltena and Wereilu. The JJA rainfall had significantly increasing trends for all stations except Mersa. Interestingly, there was no significant rainfall during January, March, April, May, July and December in all stations. This implies that the rainfall trend over the study period showed different trends for different stations at the different stations in the study area.

### Innovative trend analysis of annual and seasonal rainfall

**Fig 10:**
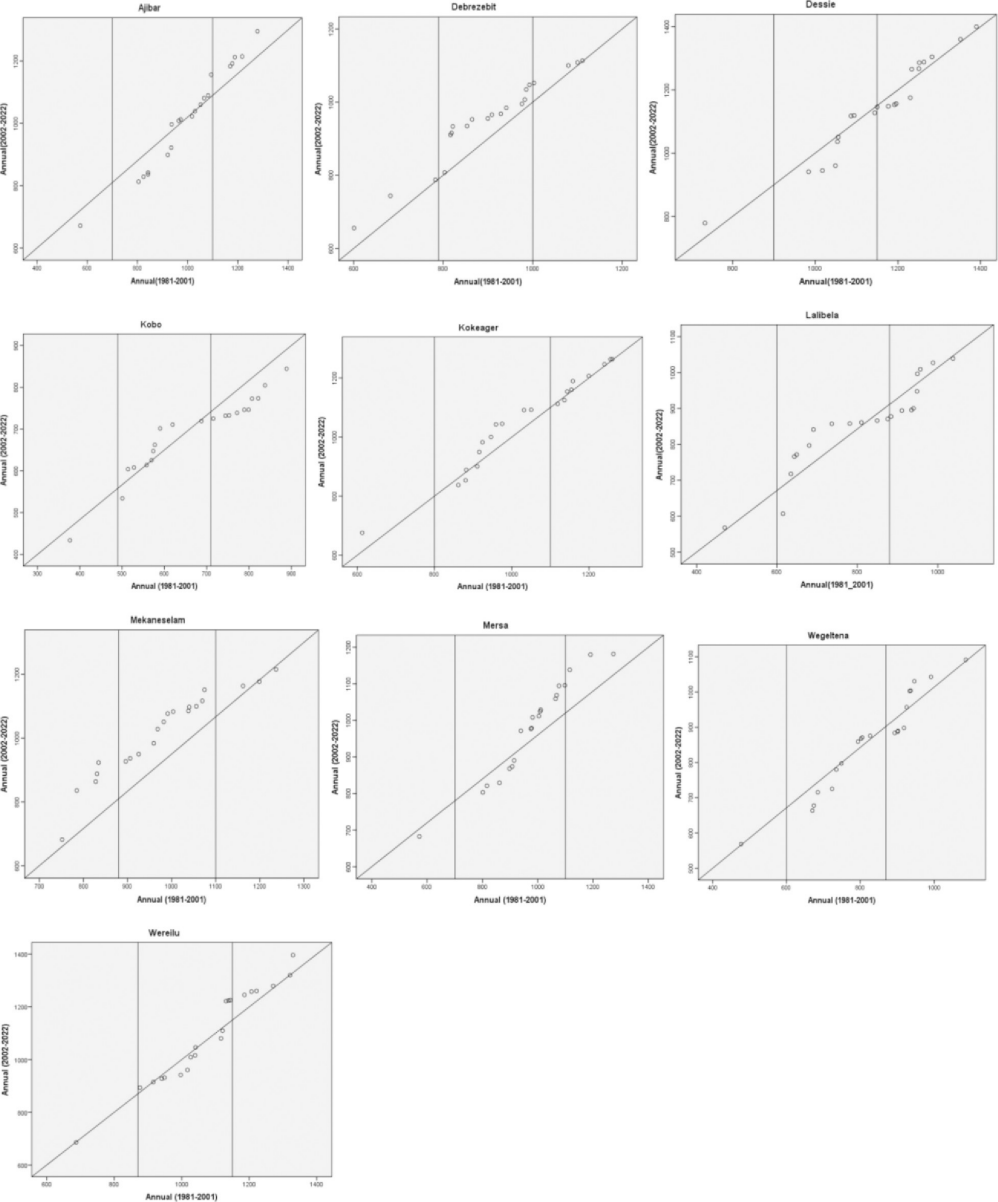
Innovative trend analysis of annual rainfall (1981–2022).

The ITA results for the annual rainfall in different stations are shown in Fig 10. The low magnitude values of the annual rainfall showed a declining tendency in the stations of Ajibar, Kobo, and Mersa, but no trend in Lalibela and Wereilu. The leeward side of the main rainy season is home to kobo and mersa. The low magnitude values for the other study sites show a rising tendency. Dessie, Wegeltena, and Wereilu all have declining trends in their medium magnitude values. Conversely, the yearly rainfall trend in Debrezebit, Kobo, and Lalibela was on the rise. The trends for the two separate values of the yearly rainfall’s medium magnitude are different in Ajibar and Mersa. There is spatial variation in the rainfall patterns in the research area, as seen by the varying trends for different stations in the various annual rainfall magnitudes. The research area’s stations displayed varying trends in relation to the high yearly rainfall. In this regard, there was an increasing tendency in high value yearly rainfall in Ajibar, Debrezebit, and Mersa. The primary rainy system’s leeward ward, Kobo, has a declining trend in its high value annual rainfall. Within the high annual rainfall levels, Dessie and Lalibela showed both increasing and declining patterns of various values. Mekaneselam did not demonstrate any discernible patterns in the high yearly rainfall values.

**Fig 11:**
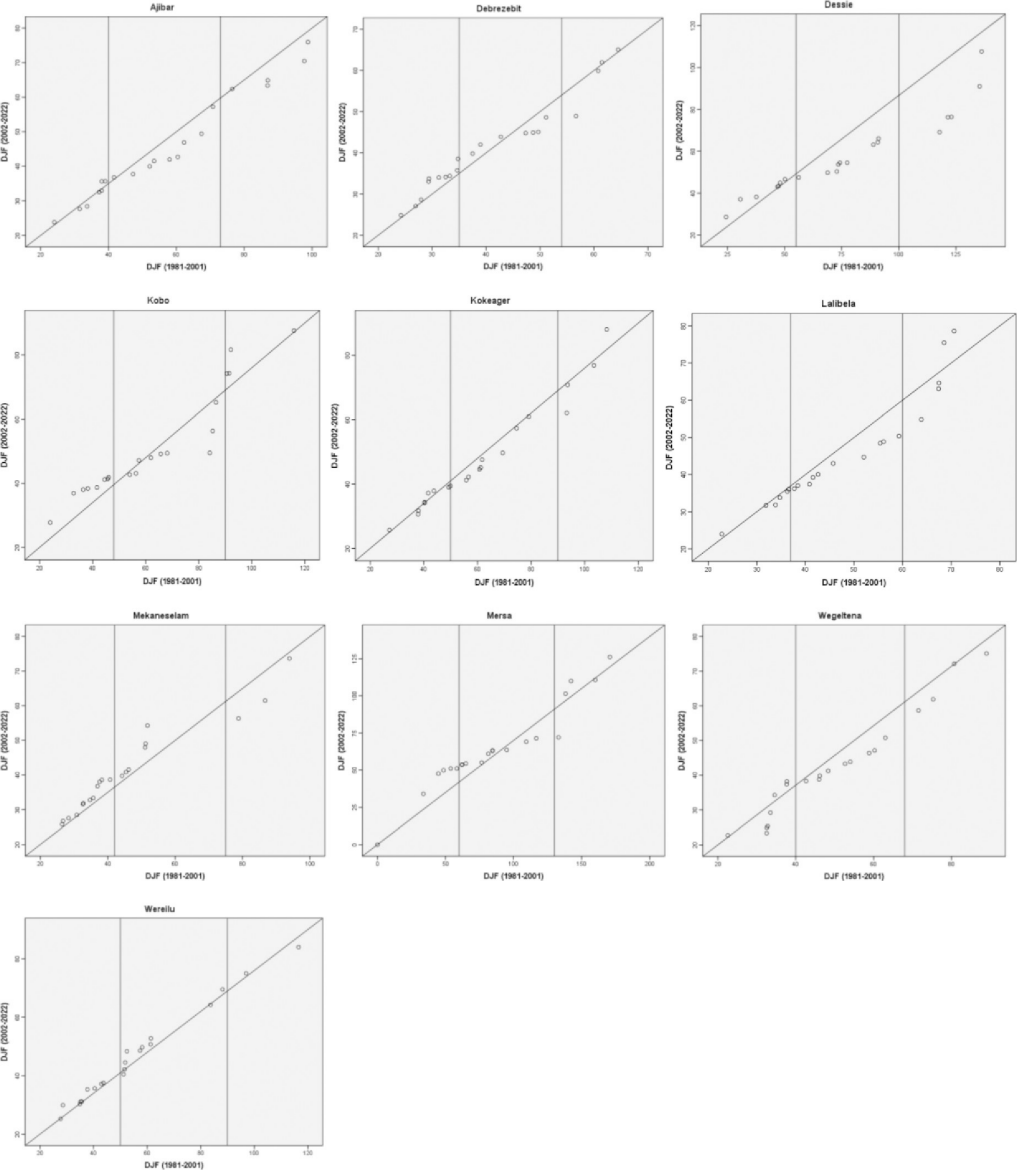
Innovative trend analysis of DJF rainfall (1981–2022).

The ITA results of the DJF rainfall trends over the research period are displayed in Fig 11. The Figure shows that, for Debrezebit, Dessie, Kobo, and Mersa stations, there is an increasing tendency shown by the low magnitude values of DJF rainfall; however, for Kokeager and Wereilu, there is no discernible trend. The low magnitude values of the DJF rainfall indicated an increasing tendency at the other study sites. For the stations in Mekaneselam and Wereilu, the medium magnitude values likewise revealed an increasing trend. Conversely, the yearly rainfall pattern in Ajibar, Dessie, and Wegeltena was declining. There is regional diversity in the rainfall patterns in the research area, as seen by the varying trends for different stations in the various annual rainfall magnitudes. The research area’s stations displayed varying trends in relation to the high yearly rainfall. As a result, while certain areas exhibited a declining trend, Lalibela and Mersa displayed a mixed trend of high DJF rainfall values. This implies that over the study period, the DJF rainfall in the Wolloo area has been decreasing.

**Fig 12:**
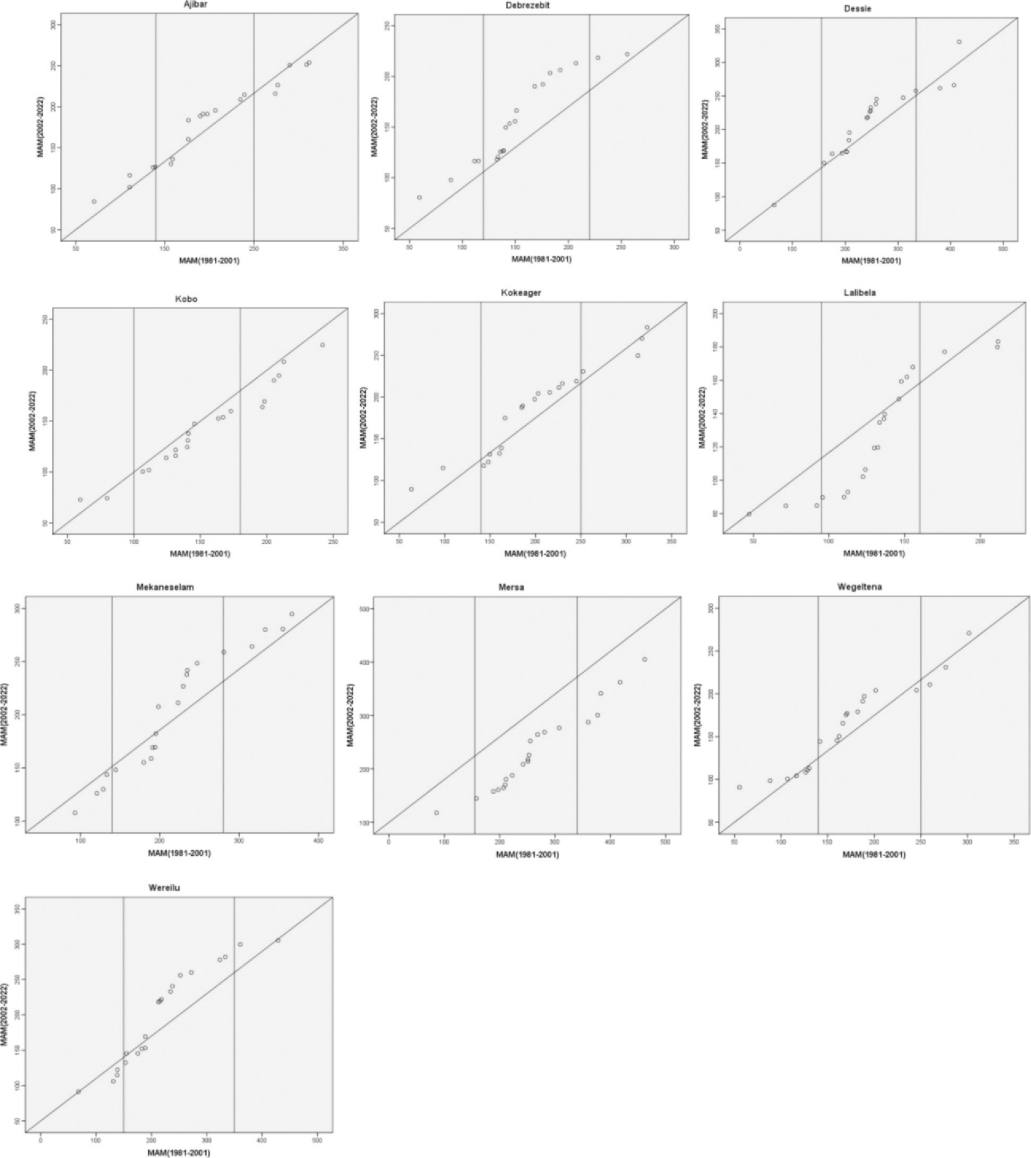
Innovative trend analysis of MAM rainfall (1981–2022).

The MAM rainfall (1981–2022) innovative trend analysis for ten meteorological stations in the research area is depicted in Fig 12. The Mekaneselam, Mersa, and Lalibela stations showed a decreasing trend, while Ajibar, Kokeager, and Wegeltena stations showed a growing trend, according to the low magnitude values of the MAM rainfall. The leeward side of the main rainy season is home to kobo and mersa. The low magnitude values for the other stations show inconsistent trends, with both an increase and a decrease in the low magnitude range’s distinctive values. For the Kobo and Mersa stations, the medium magnitude values likewise revealed a tendency toward decline. The medium magnitude value in MAM rainfall trend, however, was increasing in Ajibar, Debrezebit, Dessie, and Wegeltena. Regarding the diverse values of the medium magnitude of MAM rainfall, the other stations exhibit varying tendencies. For each of the stations in the research area, there were distinct trends in the high magnitude values of MAM rainfall. The MAM rainfall trend was increasing in Debrezebit, Mekaneslam, and Wereilu. High value MAM rainfall showed a declining trend in Ajibar, Kobo, Lalibela, and Mersa. Within the high MAM rainfall levels, Dessie, Kokeager, and Wegeltena displayed both growing and declining patterns of various values. There is regional diversity in the rainfall trends in the research area, as evidenced by the varying trends for different stations in the various magnitudes of the MAM rainfall.

**Fig 13:**
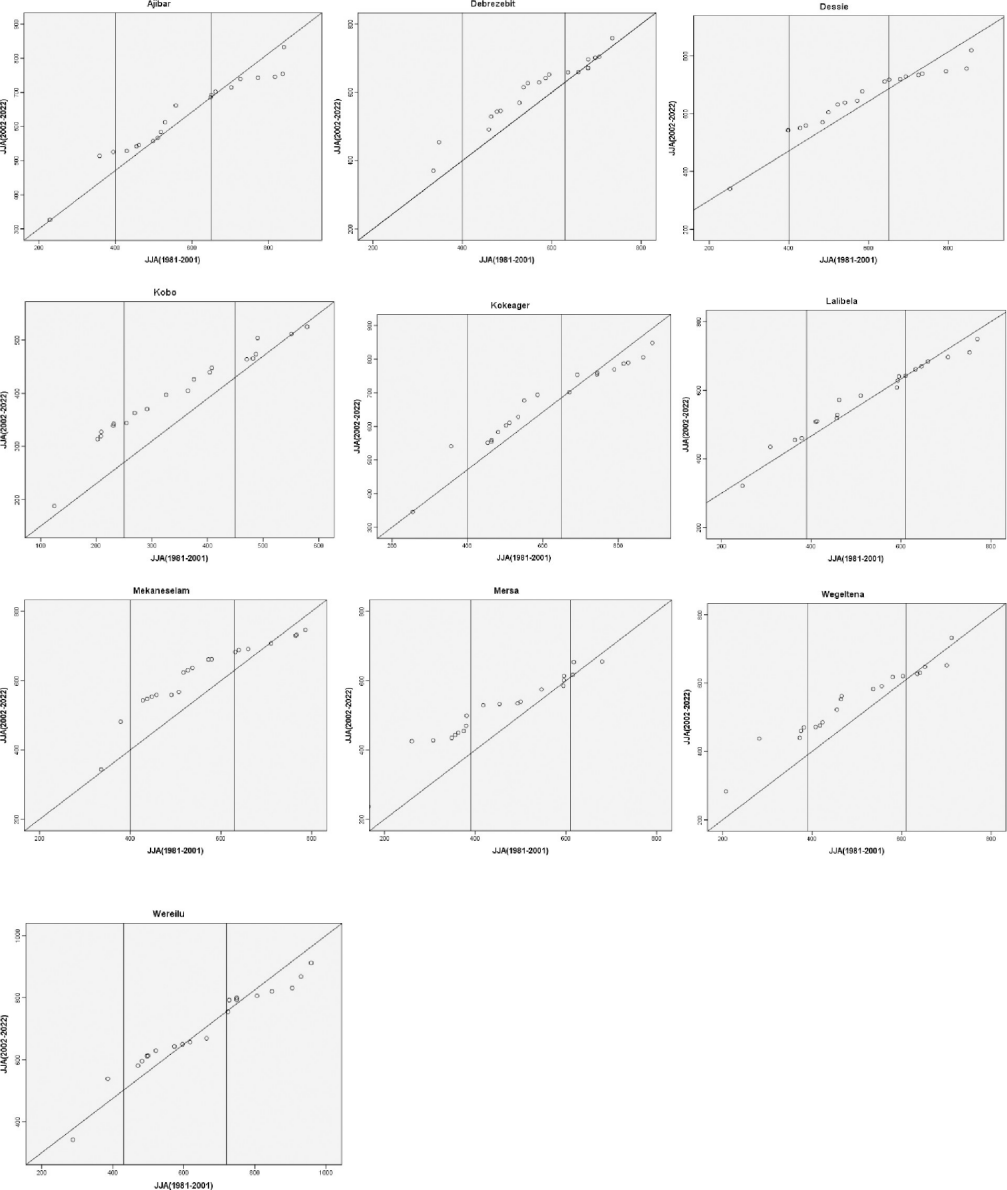
Innovative trend analysis of JJA rainfall (1981–2022).

Fig 13 shows the unique trend analysis of JJA rainfall (1981–2022) for ten meteorological stations in the research area. In the low magnitude values of the JJA rainfall, the Ajibar, Debrezebit, Kobo, Mersa, and Wegeltena stations all displayed an increasing trend, while no station displayed a falling trend. The discriminative value in the low magnitude range increases and decreases in Lalibela and Wereilu, respectively, exhibiting contradictory tendencies. An increasing trend was evident in the medium magnitude values of the JJA rainfall for Ajibar, Debrezebit, Dessie, Kobo, Kokeager, Lalibela, Mekaneselam, Mersa, and Wegeltena. This result goes with the MK test result in Table 5. No station showed a decreasing trend in the medium magnitude readings while Wereilu had a mixed tendency and that. There were distinctive patterns in the data for every station in the study area. The high magnitude values of JJA rainfall showed clear trends for every station in the study area. Kobo saw an increase in JJA rainfall while Kokeager and Ajibar saw a decline. Debrezebit had no tred, while Dessie, Wereilu, and Debrezebit showed varied trends in the JJA rainfall. The distinct patterns for different stations in the various magnitudes of the JJA rainfall demonstrate the regional variety in the rainfall trends in the research area. The JJA rain fall in the research area is generally trending upward.

**Fig 14:**
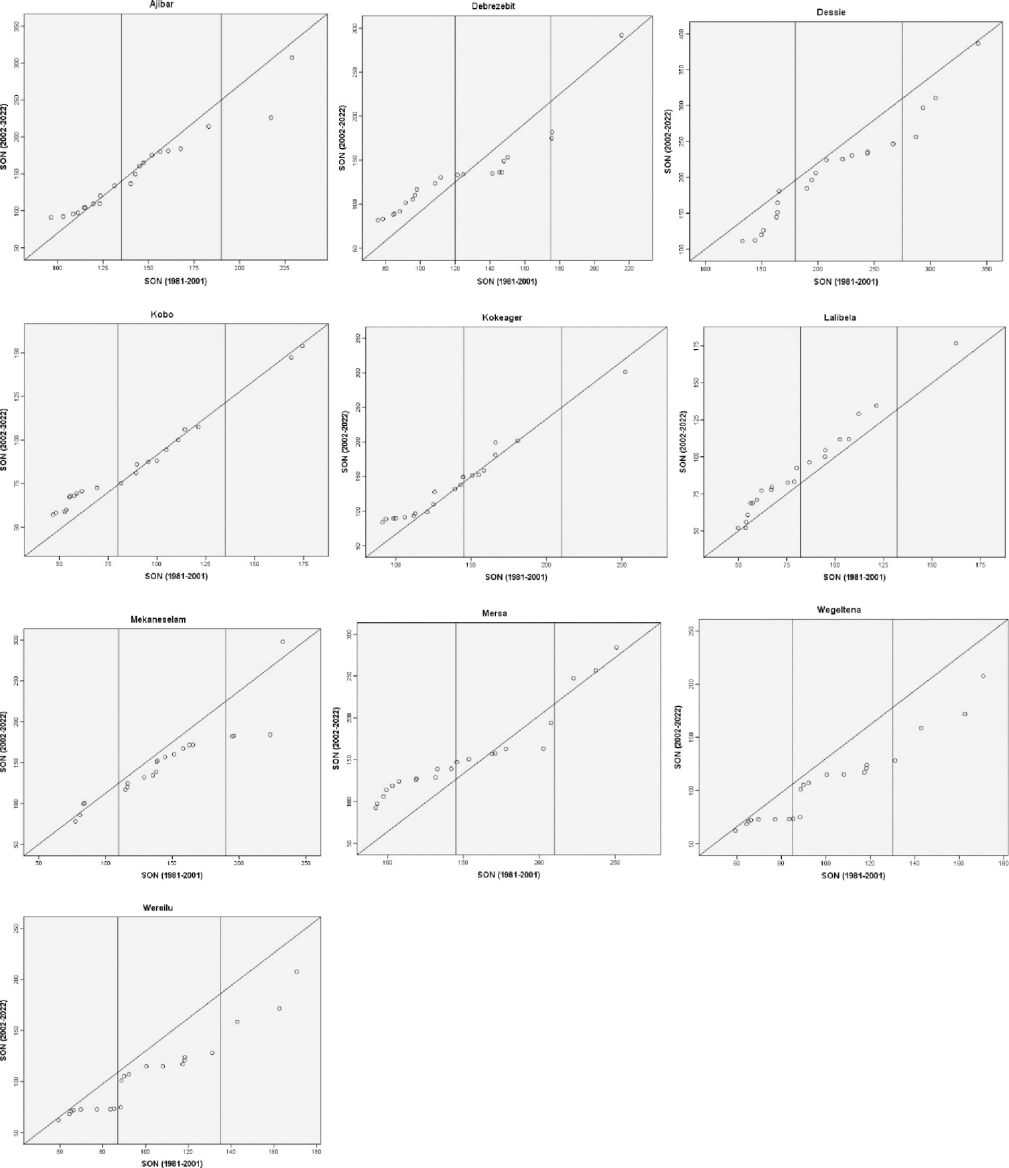
Innovative trend analysis of SON rainfall (1981–2022).

Fig 14 shows the trend analysis of SON rainfall (1981–2022) for the meteorological stations in the research area. In the low magnitude values of the SON rainfall, Debrezebit, Kobo and Mersa stations displayed an increasing trend, while Dessie, Wegeltena and Wereilu displayed a decreasing trend. The distinctive values in the low magnitude in Mekaneselam exhibited mixed trends. A growing trend was evident in the medium magnitude values of the SON rainfall for Lalibela. Debrezebit, Mekaneselam,Wereilu and Wegeltena showed a decreasing trend in the medium magnitude value of this season while Kobo had no trend. There were distinctive patterns in the data for every station in the study area. The high magnitude values of SON rainfall showed clear trends for every station in the study area. Debrezebit and Mersa saw an increase in SON rainfall while others were in the decreasing trend. The distinct patterns for different stations in the various magnitudes of the seasonal rainfall demonstrate the spatiotemporal variation in the rainfall trends in the research area.

## Discussion

The study divulges that the distribution of rainfall in the study area is affected by variability in monthly and seasonal rainfall as well as orographic effects. The eastern part of the area, which slopes down to the rift valley floor, experiences less precipitation overall and higher variability. This result is consistent with recent research that found a similar high degree of variability in monthly rainfall in northern Ethiopia. These temporal and spatial patterns are likely caused by a combination of factors, including local topography. Similar findings have been reported in Konso, southern Ethiopia, highlighting the importance of local topography in influencing the amount and distribution of rainfall [38, 39]. The same study also mentioned that the global climate shift and regional climate dynamics of the El Niño Southern Oscillation might also be possible drivers of rainfall variability. The standardized annual rainfall anomaly captured the spatial and temporal variability of rainfall, showing a high spatiotemporal anomaly with significant monthly and seasonal rainfall variability. These results are consistent with earlier research that found that rainfall in Belg had a higher coefficient of variation (CV) than rainfall in *Kiremt* in north-central Ethiopia [40].

The results of the SRA and CV analysis are consistent with the findings of previous drought years in East Africa, which have been attributed to regional climate-controlling factors and events like ENSO. For instance, Ethiopia experienced one of its deadliest droughts between 1984 and 1985, leading to the death of millions of people, particularly in the northern region, such as in Wollo [41]. The study reveals that 1984 was the driest drought year ever recorded. At the national level, the frequency of drought incidents has also been on the rise. According to a report, there has been a surge in the frequency of drought occurrences during the *Belg* season. Similarly, the *Belg* rains failed in Wollo in 1998, causing the loss of cereals and pulse harvests. According to the validation of the gridded dataset, the estimates from gridded datasets were found to be more closely matched to the station data in areas with lower altitudes (like Kobo) than those in higher altitudes (like Lalibela). This finding is consistent with the results of a previous study conducted by [24].

The *Kiremt* season (JJA) rainfall has shown an upward trend in both the MMK and the ITA methods, which is consistent with the findings of [42–44]. Similarly, [45, 46] have documented a statistically significant increase in monthly precipitation in the upper Blue Nine Basin of Ethiopia from 1981 to 2020. However, [47] reported a decrease in *Kiremt* season rainfall in the Choke Mountains of Ethiopia’s north-central highlands. Similar trends have been observed in reductions of rainfall during Ethiopia’s Belg season (MAM) [18]. A study conducted in northern Ethiopia by [42] also revealed a trend towards an increase in drought years and a decrease in *Belg* rainfall amount. Conversely, [48, 49] reported that there was no statistically significant trend in the mean rainfall observed in Ethiopia during any season between 1960 and 2006. The findings indicate that the results of research on the trends and variability of climate in the Wollo area are mixed and inconsistent, despite the increasing frequency of droughts and other climatic disasters in the study area. The trends are consistent with previous studies; for instance, [22, 50] reported a decreasing trend in rainfall in northeastern Ethiopia. Consequently, the study revealed notable fluctuations in rainfall during the observation period.

### Conclusion

The aim of the study was to investigate changes in rainfall patterns in the Wollo area between 1981 and 2022. The research involved analyzing monthly, seasonal, and annual rainfall data using non-parametric statistical indices. To conduct the study, an advanced web-based cloud computing platform called GEE was used. Data validation and calibration for the analysis of distribution, variability and trends at different temporal scales in the study area were performed by using different techniques. In this study, systematic biases from the gridded data were minimized linear scaling techniques. To assess the significance of differences between the observed and gridded data sets, different error matrices like MAPE and NRME were employed. Moreover, the effects of serial correlation in the trend analysis were assessed by calculating the lag-1 autocorrelation coefficient.

Over the study period, there were noticeable changes in rainfall patterns in the Wollo area, including changes in timing and location. Rainfall increased during the Kiremt (JJA) and Meher (SON) seasons while decreasing during the Belg (MAM) and Bega (DJF) seasons, indicating a high variability in rainfall between seasons in the Wollo area. The study also identified several extreme drought years, including 1984, which helped in understanding the trends and variability in rainfall in the area. The eastern and northeastern regions of the study area exhibited an increase in rainfall during the *Kiremt* (JJA) season. The JJA season is known for the increasing trend of rainfall as revealed by the MMK and the ITA trend analysis. The study produced maps that show monthly and annual rainfall trends, as well as spatial-temporal variations. These maps can be useful in creating spatially explicit models, which can help identify domains for adaptation technologies based on crop trials conducted under various rainfall regimes.

The results of the study can be used to create appropriate agricultural policies and practices to tackle current and future climate change. Satellite-based rainfall data is an essential source of information for determining the distribution of rainfall in areas where rainfall data is either scarce or unavailable. However, due to the combination of complex topography and limited coverage of observation stations, it is challenging to understand the patterns, trends, and variability of rainfall in the Wollo area. Accurately assessing rainfall dynamics in a heterogeneous topography based on satellite-based rainfall products remains a significant challenge for the scientific community. To gain a better understanding of the rainfall patterns in the area, an in-depth and context-specific analysis would be helpful.

## Supporting information

S1 Data (XLSX)
